# Exposure of pigs to glyphosate affects gene-specific DNA methylation and gene expression

**DOI:** 10.1016/j.toxrep.2022.02.007

**Published:** 2022-03-07

**Authors:** Knud Larsen, Thomas Bové Christensen, Ole Højberg, Martin Tang Sørensen

**Affiliations:** a Department of Molecular Biology and Genetics, Aarhus University, C.F. Møllers Allé 3, DK-8000 Aarhus C, Denmark; b Department of Animal Science, Aarhus University, Blichers Allé 20, DK-8830 Tjele, Denmark

**Keywords:** APEX1, Apurinic/Apyrimidinic Endodeoxyribonuclease 1, BER, base excision repair, CDKN1A, Cyclin Dependent Kinase Inhibitor 1A, DNMT1, DNA methyltransferase 1, DNMT3A, DNA methyltransferase 3A, DNMT3B, DNA methyltransferase 3B, EDTA, Ethylenediaminetetraacetic acid, ELISA, enzyme-linked immunosorbent assay, IL18, interleukin 18, TET3, Tet methylcytosine dioxygenase 3, TF, Transcription factor, UNG, Uracil DNA Glycosylase, Cancer, DNA methylation, Gene expression, Glyphosate, IL18, Pig, *Sus scrofa*, TET3

## Abstract

Glyphosate (*N*-(phosphonomethyl)glycine) is a broad-spectrum systemic herbicide and crop desiccant. Glyphosate has long been suspected of leading to the development of cancer and of compromising fertility. Herbicides have been increasingly recognized as epigenetic modifiers, and the impact of glyphosate on human and animal health might be mediated by epigenetic modifications. This article presents the results from an animal study where pigs were exposed to glyphosate while feeding. The experimental setup included a control group with no glyphosate added to the feed and two groups of pigs with 20 ppm and 200 ppm of glyphosate added to the feed, respectively. After exposure, the pigs were dissected, and tissues of the small intestine, liver, and kidney were used for DNA methylation and gene expression analyses. No significant change in global DNA methylation was found in the small intestine, kidney, or liver. Methylation status was determined for selected genes involved in various functions such as DNA repair and immune defense. In a CpG island of the promoter for IL18, we observed significantly reduced DNA methylation for certain individual CpG positions. However, this change in DNA methylation had no influence on IL18 mRNA expression. The expression of the DNA methylation enzymes DNMT1, DNMT3A, and DNMT3B was measured in the small intestine, kidney, and liver of pigs exposed to glyphosate. No significant changes in relative gene expression were found for these enzymes following dietary exposure to 20 and 200 ppm glyphosate. In contrast, a significant increase in expression of the enzyme TET3, responsible for demethylation, was observed in kidneys exposed to 200 ppm glyphosate.

## Highlights

• A large animal study with exposure of pigs to glyphosate is presented here.
• Pigs were exposed to 20 ppm and 200 ppm in the diet.
• No significant changes in global DNA methylation was observed.
• A significantly reduced DNA methylation was found in the porcine IL18 promoter.
• A significant increase in TET3 expression was seen in porcine kidneys exposed to 200 ppm glyphosate.

## Graphical Abstract

**Figure:**
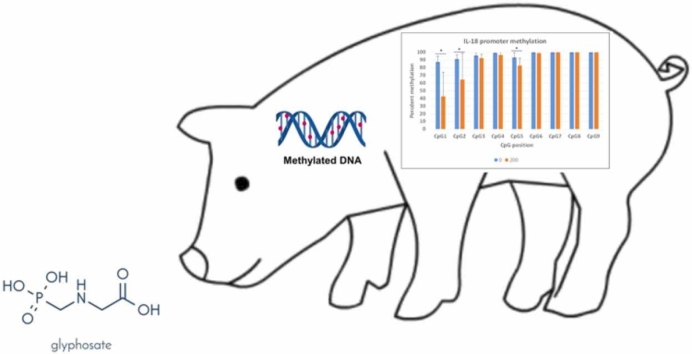

## 1 Introduction

Glyphosate (*N*-(phosphonomethyl)glycine) is a broad-spectrum systemic herbicide and crop desiccant that functions by inhibiting the plant enzyme 5-enolpyruvylshikimate-3-phosphate synthase, an enzyme involved in the synthesis of the aromatic amino acids tyrosine, tryptophan, and phenylalanine. Glyphosate is widely used for effective weed control. Glyphosate is also commonly used in the cultivation of crops, either for desiccation of grain, or for the control of weeds in the cultivation of crop plants genetically modified to be resistant to glyphosate. These uses can result in glyphosate residues in animal feed [1].

Xenobiotics can induce epigenetic modifications, which can lead to the development of chronic diseases. Epigenetic modifications include changes in global methylation and gene-specific methylation within promoters. The modulation of DNA methylation can affect gene expression, and this may have important consequences for cells and organisms [2], [3], [4]. Given that glyphosate is globally one of the most widely used herbicides [5], it is crucial to determine the epigenetic properties of this component; these properties can be measured both in vitro and in whole organisms. Glyphosate has long been suspected of leading to the development of cancer and of compromising fertility.

A study of the epidemiological literature up to 2021 was conducted to evaluate whether glyphosate increases the risk of developing cancer in humans. This study found no consistent pattern of positive associations between cancer and glyphosate exposure [6]. A literature survey of the potential role of glyphosate in cancer development revealed 37 significant cases of tumor findings and showed consistency across studies in the same animal species and sex for many of the tumors [7]. The strongest evidence shows that glyphosate causes the development of many different cancer types in mice and rats (reviewed in Portier [7]). However, despite these results, there is still a disagreement about the carcinogenic effect of glyphosate. Human epidemiological studies have shown a possible link between glyphosate and non-Hodgkin’s lymphoma [8], [9]. Other studies have demonstrated an increased risk of developing non-Hodgkin's lymphoma with prolonged exposure to glyphosate [10], [11]. In contrast, the largest and the most recent epidemiological survey, the independent 2018 National Cancer Institute-supported Agricultural Health Study, has not revealed any association between glyphosate-based herbicide exposure and cancer development [12]. Cancer and other chronic diseases, such as type II diabetes, can be caused by interactions between genetic, epigenetic, environmental and lifestyle factors. Epigenetic modifications, such as DNA methylation, is influenced by both environmental factors and lifestyle factors. Exposure to certain factors, e.g. in agricultural environments, can affect DNA methylation of specific genes related to asthma and allergies [13], [14], [15], [16]. Several herbicides are known to affect epigenetics. An example is the herbicide diuron, which affects the methylome of Pacific oyster [17]. Herbicides can also affect gene expression and activity. This is exemplified by the effect of glyphosate on the expression of the estrogen receptors ERα and ERβ on human breast cancer cells [18]. The changed expression of the estrogen receptors induces cell proliferation, which my lead to development of cancer. Other studies have also shown that glyphosate affects the activity of ERα in cancer cells [19], [20], [21].

Recently, Kubsad et al. [22] and BenMaamar et al. [23] reported a relationship between glyphosate, sperm DNA methylation, and transgenerational inheritance of pathologies and disease.

Glyphosate affects both the distribution of the degree of DNA methylation in the chromatin in animals and plants [24]. Methylation levels are positively correlated to the severity of the herbicide injury [24], [25]. Glyphosate exposure also changes DNA methylation in mammalian cells. Kwiatkowska et al. [26] found a reduction in DNA methylation in human peripheral blood mononuclear cells exposed to glyphosate. Similar results of reduced global DNA methylation were shown by Wozniak et al. [27]. In their study, they also reported that glyphosate affected the expression of genes involved in the regulation of cell cycle and apoptosis, e.g. P16 and TP53, BCl2, CCND1, and P21 [27], [28].

The aim of the present study is to determine whether there are any epigenetic effects of glyphosate on pig genes, particularly in terms of DNA methylation and gene expression. Hence, this study is a whole-animal study of glyphosate exposure, as opposed to a cell culture study. The biological samples used in this study were obtained from a project that examined whether glyphosate affects the health, productivity, and mineral status of weaned pigs [29] and the composition of the bacterial population in the gut [30]. Until now, studies of the potential effects of glyphosate on livestock, including pigs, are scarce and mainly report in vitro examinations. We therefore used organs from a study with pigs exposed to glyphosate through their diet to study glyphosate’s possible effects on DNA methylation and gene expression. We present evidence that glyphosate induces gene-specific DNA hypomethylation in the IL18 gene promoter in the liver of pigs exposed to glyphosate. Furthermore, we observed an increase in expression of the DNA demethylating enzyme TET3 in pig kidneys. Thus, the present study provides evidence that glyphosate can induce gene expression changes and gene-specific epigenetic effects in pigs.

## 2 Materials and methods

### 2.1 Ethics

The biological material used in this study was retrieved from two successive animal experiments conducted by Krogh et al. [29]. Protocols for housing and feeding in these experiments were approved by The Danish Animal Experiments Inspectorate, as described [29].

### 2.2 Animals

A total of 104 crossbred (Duroc × [Landrace × Yorkshire]) pigs from 13 litters were weaned at 28 days of age. One litter per week was included in the experiment, with eight piglets from each litter (four females and four males/barrows) were randomly selected to be included in the experiment. The four piglets of each gender were ranked by body weight and assigned to one of four experimental treatments (n = 26 pigs/treatment) based on a predefined randomization procedure to randomize piglet weaning weight among treatments. Three of the four treatments were included in the present investigation, namely the control treatment (CON) without glyphosate added to the diet and two treatments with planned glyphosate contents of 20 ppm and 200 ppm added to the diet in the form of isopropylamine salt of glyphosate. Half of the pigs were slaughtered after nine or 10 days of treatment, while the other half were slaughtered after 35 days of treatment. In the present investigation, we used samples from the small intestine, liver, and kidney obtained from the pigs slaughtered after 35 days of treatment. The samples were stored at − 20 °C until the time of analysis. Details on housing and diet formulation were described by Krogh et al. [29].

### 2.3 Isolation of DNA and RNA from pig samples

Pig DNA was isolated using a salt precipitation method. Tissue from pig organs was homogenized using a tissuelyzer. For 25–50 mg homogenate, 300 µL lysis buffer (50 mM Tris pH 8, 100 mM EDTA, 100 mM NaCl, 1% SDS) and 30 µL (10 mg/mL) proteinase K (Bionordika) were added, and the homogenate was then incubated at 55 °C for 12–18 h. Next, the sample was vortexed briefly, and 180 µL 6 M NaCl were added, and the composition was mixed vigorously. The mixture was centrifuged at 15,000 rpm for 15 min at 4 °C, and approximately 500 µL of supernatant was transferred to a microcentrifuge tube. Isopropanol (1:1) was added, and the sample was mixed and subsequently centrifuged at 15,000 rpm for 10 min at 4 °C. The resulting pellet was washed with 400 µL 70% ethanol. After centrifugation at 15,000 rpm for 10 min at 4 °C, the ethanol was removed, and the pellet was resuspended in 100 µL 1X TE and 1 µL (10 mg/µL) (Sigma) RNase and then incubated overnight at 4 °C. The DNA concentration was measured using Nanodrop (DeNovix). The DNA quality was checked for all DNA samples using agarose gel electrophoresis with GelRed (Novus Biologicals). Aliquots of the DNA were stored at − 20 °C.

The RNA was isolated from the pig organs using two different methods. For some samples, we used the TRIzol™ Plus RNA Purification Kit and Phasemaker™ Tubes complete system (Invitrogen). TRIzol purification of RNA was performed according to the manufacturer’s manual. For the remaining samples, we used the PureLink RNA Mini Kit (Ambion). For this kit, we followed all the manufacturer’s recommendations as guidelines for RNA purification. The concentration of purified RNA preparations was measured using Nanodrop (DeNovix). The RNA quality was checked for all RNA samples using agarose gel electrophoresis with GelRed (Novus Biologicals), which allowed visualization of the distinct 28 S and 18 S bands and checked for potential DNA contamination. The RNA preparations were stored at − 80 °C.

### 2.4 Bisulfite conversion of DNA

Bisulfite conversion of DNA samples was performed with the EZ DNA Methylation-Gold™ Kit (Zymo Research/Nordic BioSite) to detect DNA methylation [31]. The method relies on bisulfite conversion of unmethylated cytosine (C) into uracil (U) while at the same time conserving originally methylated CpGs. If a cytosine is methylated, the methylation protects the base from bisulfite conversion and remains unchanged. Bisulfite conversion was carried out according to the manufacturer’s protocol. The concentration of bisulfite-converted DNA preparations was measured using Nanodrop (DeNovix). The eluted DNA was stored at − 20 °C for short-term storage and − 80 °C for long-term storage.

### 2.5 Measurement of global methylation

Global methylation was measured by means of DNA quantification using 5-mC monoclonal antibodies in an enzyme-linked immunosorbent assay (ELISA)-like reaction with the Methylflash Methylated DNA Quantification Kit (Epigentek). The DNA methylation levels were calculated in relation to the methylated control DNA and expressed as a percentage of methylated DNA. The DNA (100 ng) isolated from porcine small intestines, livers and kidneys was used for analysis. Each sample was analyzed in technical duplicate. The calculation of 5-mC amount was performed with the use of a standard curve created using defined dilutions of methylated genomic DNA.

### 2.6 Bisulfite sequencing and estimation of DNA methylation rate

Genomic DNA from different pig organs was isolated and bisulfite-treated using the EZ DNA Methylation Kit (Zymo Research) following the manufacturer's instructions. Gene-specific primer sets were used in PCR amplification of defined promoter fragments from bisulfite-treated DNA (Table S1). The PCR amplification primers and sequencing primers were designed using MethPrimer software [32].

Identification of the methylation status relied on the ratio between C and T peaks in a chromatogram visualized with sequence analysis programs like Chromas and CodonCode. The ratio between peak heights of C and T and a given CpG site was quantified using the following equation: methylation percentage = (C/(C+T) * 100) [33], [34].

### 2.7 Gene expression analysis

Porcine DNMT1, DNMT3A, DNMT3B, TET3, CASP3, and IL18 mRNAs expression were determined through real-time RT-PCR analysis. Porcine small intestines, livers, and kidneys were included in the analysis. Eight individual biological samples of each type of tissue and GAPDH were used as a reference gene to determine individual transcripts’ expression.

Primers and probes used in the expression analysis were identified by the aid of the Probe Finder web tool (www.roche-applied-science.com) and the Human Probe Library. When possible, gene-specific primers were constructed to span neighboring exon junctions. The names and sequence of the oligonucleotide primers and probes are shown in Table S1. A quantitative RT-PCR test was performed as previously described [35]. PCR products were examined by agarose gel electrophoresis and ethidium bromide staining (data not shown). Expression data were analyzed as previously described [35].

### 2.8 Bioinformatic tools

MethPrimer was used to predict and identify CpG islands in the various selected gene promoter sequencers (http://www.urogene.org/methprimer) [32]. Predicted bisulfite-conversion-based methylation PCR primers were used in the determination of the gene-specific DNA methylation rate (see paragraph 2.6).

### 2.9 Statistical analysis

For the expression analysis, the mean value was obtained for each of three individuals per litter analyzed in technical triplicate. The individuals were selected from three independent litters. Possible influence from the litter on the resulting statistics was tested and refuted. Eight pigs from each experimental group were included in the statistical analysis. Statistical analysis was conducted using the Mann-Whitney test (for samples with distributions departing from normality), the Student's t-test (for samples with the normal distribution), and ANOVA with the post-hoc multiple comparisons procedure (Tukey test). The differences are statistically significant when *P* values are less than 0.05.

## 3 Results and discussion

### 3.1 Glyphosate in diet and daily intake

The glyphosate content of the pigs’ diets was analyzed using the microLC-MS/MS method [36]. The analyzed diet glyphosate contents were 0.02 ppm for control and 22.0 ppm and 208 ppm, respectively for the two groups with glyphosate added to the feed [29]. Notably, glyphosate level in the control diet was not zero, indicating that glyphosate-free diets are very difficult to obtain, e.g. due to feed crop uptake from a soil pool or from airborne glyphosate-containing aerosols through spray application in neighboring fields or both [37].

The average body weight of the pigs in the week before slaughter was 20.5 kg and average daily feed intake in the same period was 1230 g. Based on the average body weight and feed intake in each of the three groups, the average daily intake of glyphosate in the week before slaughter was 0.001 mg per kg body weight for control pigs and 1.31 and 12.4 mg, respectively for the two groups with glyphosate added to the feed.

### 3.2 Global DNA methylation

The best and the most frequent epigenetic event is the aberrant methylation of DNA. It occurs due to changes in the activity of DNA methyltransferases (DNMTs) and the addition of methyl groups to cytosine nucleotides in the CpG position, which regulate chromosomal stability and gene expression; DNA methylation plays an important role in natural growth and physiological processes such as cellular differentiation, oncogenic transformation, and long-term memory formation.

**Fig. 1:**
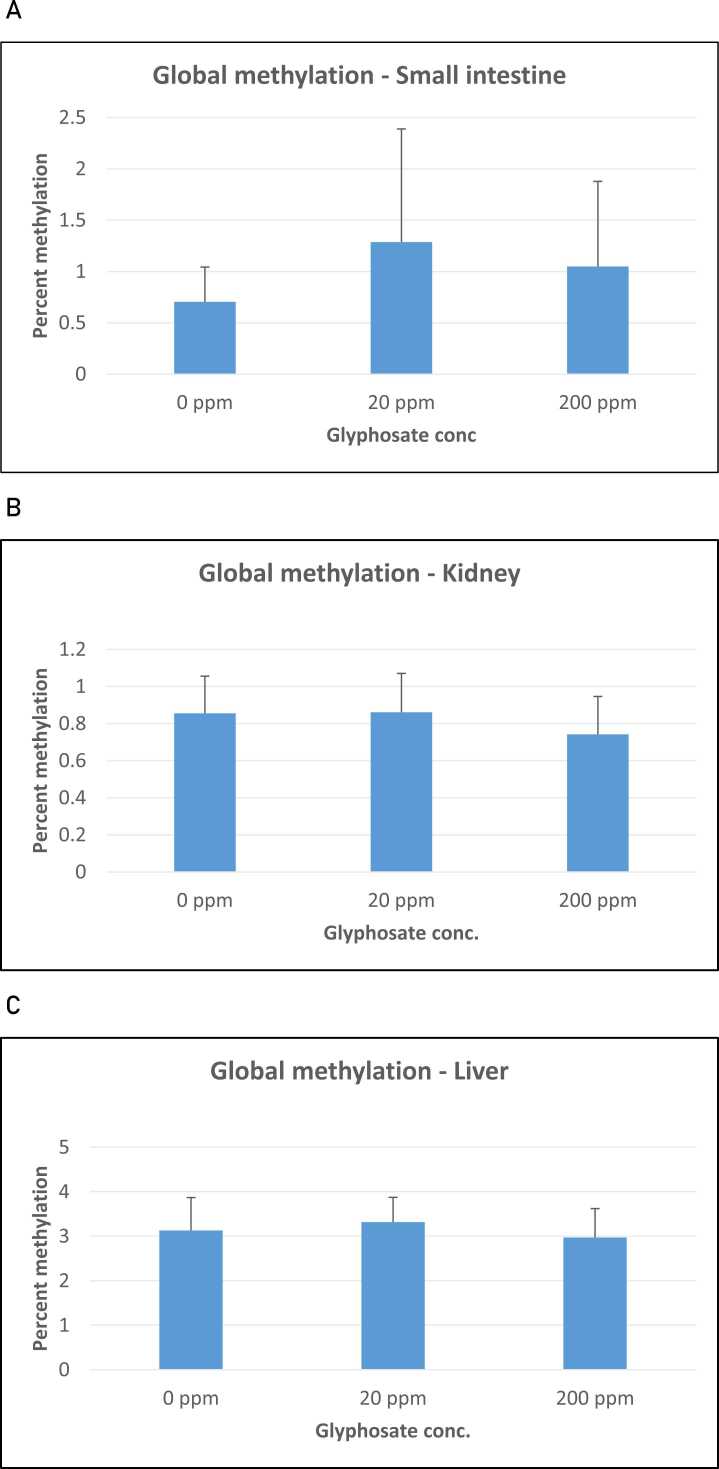
Global methylation in A) small intestine, B) kidney, and C) liver DNA isolated from control pigs not treated with glyphosate (0 ppm) and pigs exposed to 20 ppm and 200 ppm dietary glyphosate (means ± SEM), depending on dietary glyphosate concentration. Six individuals from each group were included in this study. No significant difference was observed in global DNA methylation in intestines in the control (no glyphosate added) group and the group receiving treatments of 20 ppm glyphosate.

Therefore, the global DNA methylation was determined for the three experimental groups: the group of pigs exposed to 20 ppm glyphosate, the group of pigs exposed to 200 ppm glyphosate, and the control group with no glyphosate added to their diet. Thirteen animals from each group were subjected to global DNA methylation analysis using 5-mC monoclonal antibodies in an ELISA-like reaction with the Methylflash Methylated DNA Quantification Kit (Epigentek). The DNA was isolated from small intestines, kidneys and livers, and 100 ng DNA was used in the analysis. As shown in Fig. 1, no significant changes in global DNA methylation were observed in small intestines, kidneys or livers as a result of the glyphosate treatment. However, differences in the absolute value of global DNA methylation were seen between the three organs. Whereas DNA methylation rates were around 1% (0.8%−1.2%) in the small intestines and kidney tissues, a higher value of around 3% was observed in liver tissue (Fig. 1A-C).

A global DNA methylation analysis of porcine muscle revealed a methylation percentage of approximately 4% [38]. In another bisulfite-sequencing-based study, it was shown that global CpG methylation is similar across the pig neocortex, spleen, liver, femoral muscle, and olfactory epithelium [39]. The authors detected a low rate (average of 1.7%) of non-CpG methylation in the six samples, with the exception of the neocortex (2.3%) [39]. Collectively, the observed global CpG methylation patterns of pigs featured high similarity to other mammals, including humans [39]. Similarly, Schachtschneider et al. [40] reported on global DNA methylation in six different tissues and organs from adult pigs. The results were obtained using genome bisulfite sequencing and showed low non-CpG methylation (<1%) in all non-neuronal somatic tissues as well as the heart, kidney, liver, lung, muscle, spleen, and lymph nodes.

Kwiatkowski et al. [26] conducted a study examining the effect of glyphosate in human peripheral blood mononuclear cells (PBMC) in vitro. The global methylation of blood cells exposed to glyphosate was determined with DNA quantification using 5-mC monoclonal antibodies in an ELISA-like reaction and revealed statistically significant changes in global DNA methylation (5-mC percentage) in PBMCs treated with glyphosate. In comparison with control cells, the percentage of global DNA methylation levels was significantly decreased by glyphosate at concentrations of 0.25 mM (~42 mg/liter) as well as 0.5 mM (~85 mg/liter) [26]. In contrast, we did not observe any effect from glyphosate on the global DNA methylation in the pigs exposed to 20 ppm and 200 ppm glyphosate.

Wozniak et al. [27] determined the effect of glyphosate on epigenetics in human peripheral blood mononuclear cells (PBMCs). Their results revealed a significant reduction in global DNA methylation levels in PBMCs exposed to glyphosate. Differences in DNA global methylation response to glyphosate compared to cell culture experiments and our data in this whole-animal experiment could be explained by the means of glyphosate exposure and how effects are expressed. We did not examine DNA methylation in blood samples of pigs exposed to glyphosate, and we therefore cannot rule out an effect of glyphosate on the DNA methylation of blood cells. In our experimental design, we did not include long-term exposure (>35 days) of pigs to glyphosate. Hence, we cannot rule out that long-lasting effects of glyphosate on gene expression and epigenetics alteration could appear.

### 3.3 DNA methylation rates in selected gene promoters

A small number of gene promoters were selected for the gene-specific DNA methylation analysis (Table 1). The rationale for choosing the genes and their promoters was based on their functional roles in the development of cancer either as tumor suppressors and oncogenes, such as p16, p21, TP53 and RASSF1A; as members of the base excision repair pathway (BER) NEIL1, NEIL2, OGG1, APEX1, POLB and UNG; or as modifiers of DNA (DNMT3A; Table 1). In addition, we included the genes IL18, MANF, and MAPT in order to study genes with different roles in immune response and brain functioning.

**Table 1:** DNA methylation of CpGs in promoter sequences 2000 bp 5′-upstream of transcription start site (TSS).

**Gene**	**Protein**	**CpG island (s)**	**DNA methylation**
CDKN2A	P16	4 (1^a^)	No
CDKN1A	P21	0 (1^a^)	Yes
TP53	Tumor Protein P53	3	No
RASSF1A	Ras Association Domain Family Member 1	1 (1^a^)	No
NEIL1	Nei Like DNA glycosylase 1	3	No
NEIL2	Nei Like DNA glycosylase 2	1 (1^a^)	No
OGG1	8-oxoguanine DNA Glycosylase	2	Yes
APEX1	Apurinic/Apyrim. Endodeoxyribonuclease 1	2	Yes
POLB	DNA Polymerase Beta	0 (1^a^)	Yes
UNG	Uracil DNA Glycosylase	2 (1^a^)	Yes
IL18	Interleukin 18	1	Yes
DNMT3A	DNA methyltransferase 3A	1	Yes
MANF	Mesencephalic Astrocyte Deriv. Neurotro. Fact.	1	No
MAPT	Microtubule Associated Protein Tau	1	No

** Methylation in exon 1.

a CpG island in exon 1.

Very often, a gene promoter consists of a core promoter region immediately upstream of the transcription start site and a regulatory region further 5′-upsteam containing recognition sequences for specific transcription factors (TF). DNA methylation, mostly within cytosine-guanine dinucleotides (CpGs), have the potential to modulate transcription factors binding to DNA. Although DNA methylation has long been thought to repress TF binding, a more recent model proposes that TF binding can also inhibit DNA methylation. It is therefore of relevance to identify transcription factors (TF) located within CpG islands. We have initiated our epigenetic studies by performing in silico analyses of selected gene promoters, examining both for the presence of transcription factor (TF) binding sites and for the presence of CpG islands. Both CpG islands and specific TF binding sites were identified far upstream of the TSS. Therefore, we choose to include 2000 bp upstream of the TSS.

For all genes listed in Table 1, a 2000 bp upstream sequence of the transcription start site (TSS) for porcine genes was analyzed for the presence of CpG islands using MethPrimer software. For some gene promoters, including CDKN1A encoding P21 and POLB coding for DNA polymerase beta, no CpG islands were identified within the 2000 bp sequence (Table 1). However, for both CDKN1A and POLB, a CpG island was found in the exon 1 sequence. For some of the gene promoters, no methylation of CpGs within the identified CpG island were observed; this was seen for CDKN1A, TP53, RASSF1A, NEIL1, NEIL2, MANF, and MAPT (Table 1).

**Fig. 2:**
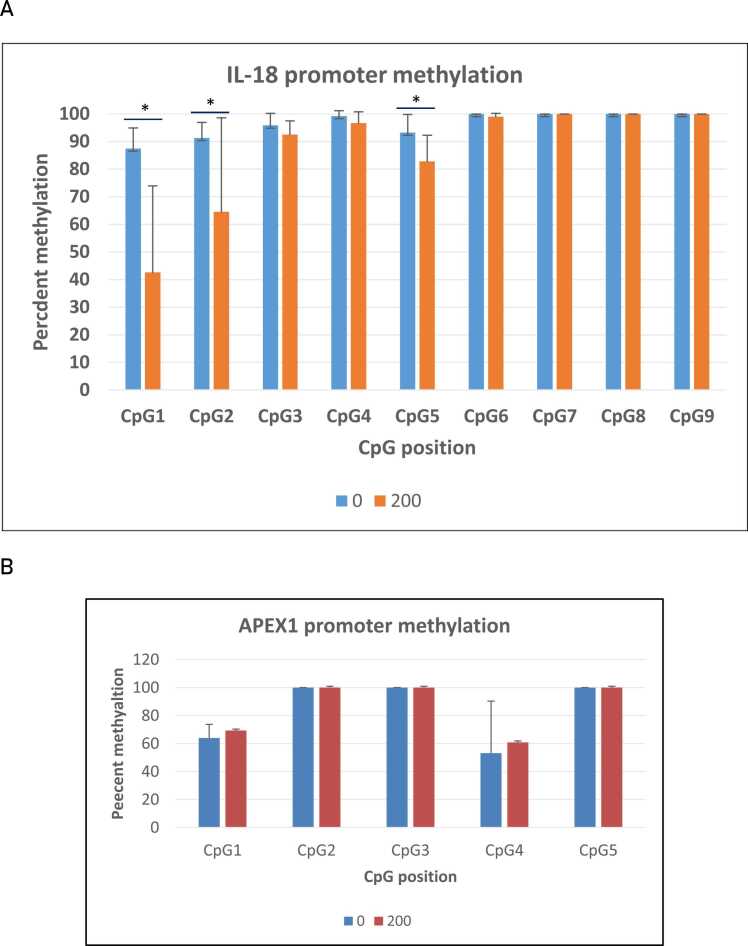
Glyphosate-induced changes of DNA methylation status in the IL18 promoter. DNA methylation was determined by bisulfite sequencing in nine CpG positions in the IL18 promoter. DNA methylation was measured in small intestine DNA (A), kidney (B) and liver (C) from pigs exposed to 200 ppm glyphosate and in a control group with untreated pigs. DNA methylation was estimated by calculating from top heights for C and T in each experimental group (n = 8 for each group). * P < 0.05.

Our in silico analysis of the porcine IL18 promoter revealed a CpG island with nine CpGs. The island is situated at positions − 200 to − 400 upstream of the TSS in the 5′-flanking region of the porcine IL18 gene. A sequence within this CpG island containing nine CpG positions was subjected to bisulfite sequencing analysis. As shown in Fig. 2A-F and Fig. S1, we observed very high methylation rates for all nine CpG positions in the DNA from porcine small intestines; for CpGs 6–9, methylation rates were close to 100% (Fig. S1). For positions CpG1 and CpG2, we observed significant reduction in DNA methylation percentages in the small intestines DNA from pigs treated with 200 ppm glyphosate (Fig. 2A and B). For CpG1, the reduction went from 87% in the control intestines to 55% in the 200-ppm-treated intestines (p = 0.0037). Similarly, significant reductions in DNA methylation were seen in CpG2 (p = 0.043) and CpG5 (p = 0.019) (Fig. 2B and E).

IL18 encodes the protein interleukin 18 which belongs to the IL-1 superfamily, and is produced mainly by macrophages. IL18 is a proinflammatory cytokine that stimulates various cell types and has pleiotropic functions. It augments natural killer cell activity in spleen cells and stimulates interferon gamma production in T-helper type I cells.

IL18 acts together with IL-12, and induces cell-mediated immunity following infection with microbial products such as lipopolysaccharide (LPS).

Expression of the human IL18 gene is controlled by two promoters: a 5′-flanking promoter and an intron 1 promoter, which are both inducible by LPS [41]. ICSBP and PU.1 are critical transcription factors for IL18 promoter activity, performing dominant roles in inducible and constitutive expression of IL18 [41]. Rusiecki et al. [42] studied the DNA methylation of the IL18 promoter in post-traumatic stress disorder (PTSD) patients. They examined a 500 bp sequence containing the 5′UTR and the coding sequence of exon 1 and 300 bp upstream of the TSS. The DNA methylation status was analyzed for three CpG positions in the promoter region. The authors demonstrated increased IL18 methylation in PTSD cases and also showed that there was a reduction in DNA methylation among non-disease controls.

APEX1 encodes apurinic/apyrimidinic endodeoxyribonuclease 1, a DNA glycosylase, which plays a central role in the cellular response to oxidative stress. The APEX1 protein functions as a DNA repair enzyme in the base excision repair (BER) pathway.

Two CpG islands were identified in the 5′-flanking region of the porcine APEX1 gene.

A sequence approximately − 1600 to − 1900 bp upstream of the TSS in the APEX1 5′-flanking region was selected for the bisulfite sequencing analysis. Differential DNA methylation was seen for the six CpGs in the analyzed APEX1 promoter sequence, with values from 50% to 100%. However, no significant changes in methylation rate were found for the six CpG positions when compared to DNA from the small intestines of the untreated control group versus the group exposed to 200 ppm glyphosate (Fig. S1).

The in silico analyses identified two CpG islands in the promoter region of UNG and one CpG island in exon 1. We selected a sequence approximately − 1200 to − 1400 bp upstream of the TSS containing six CpGs for the bisulfite DNA methylation analysis. A differential methylation rate was observed in small intestine DNA for the six CpGs, with methylation values of 80%− 100%. The highest values (100%) were seen in CpG4 and CpG6 (Fig. S2A). The lowest value was found in the CpG6 position in the intestines of pigs not exposed to glyphosate (Fig. S2A). No significant changes were seen when comparing the values for 0 ppm and 200 ppm of glyphosate. There was a tendency toward an increase in methylation ratio in the CpG6 position from 0 ppm versus 200 ppm glyphosate. At the same time, there was a very high variation in values for 0 ppm. The UNG gene encodes uracil-DNA glycosylase. This enzymes excises uracil from single-stranded DNA and mismatched double-stranded DNA, thereby releasing uracil. The UNG protein initiates the base-excision repair (BER) pathway.

We also included CDKN1A, commonly termed P21, in our methylation status analysis. The MethPrimer analysis revealed a CpG island in exon 1. This particular CpG island contains seven CpGs; all seven CpG positions display very high values of DNA methylation rates (~100%; Fig. S1B). No significant changes in methylation rate were observed for the seven CpG positions when comparing DNA from the small intestines of the untreated controls versus the groups exposed to 20 ppm and 200 ppm glyphosate (Fig. S2B).

The CDKN1A gene encodes cyclin-dependent kinase inhibitor 1A, and the CDNK1A (also named P21). The P21 protein binds to and inhibits the activity of cyclin-cyclin-dependent kinase 2 or cyclin-cyclin-dependent kinase 4 complexes, and hereby it functions as a regulator of cell cycle progression at G1. The expression of the CDKN1A gene is controlled by the tumor suppressor protein p53. The P21 protein plays a regulatory role in S phase DNA replication and DNA damage repair. Wozniak et al. [28] showed that glyphosate changes the methylation pattern of the P21 and TP53 promoters in PMBCs treated with glyphosate. The authors demonstrated a significant decrease in methylation within the P21 gene promoter in PMBCs treated with glyphosate at a concentration of 0.5 μM. In contrast, increased DNA methylation was observed in the TP53 tumor suppressor gene upon exposure to glyphosate. For other analyzed genes, such as P16*,* BCL2, and CCND1, no statistically significant changes in gene promoter methylation levels were detected.

The DNA methyl transferase (DNMT) enzymes DNMT3A and DNMT3B are responsible for de novo methylation in genomic DNA. Increased expression of DNMT3A and DNMT3B leads to the hypermethylation of tumor suppressor genes [43]. The decrease DNA methylation affects tumor suppressor gene expression and the genes involved in cell cycle, DNA repair, carcinogen metabolism, cell adherence, and apoptosis [44]. We therefore examined the rate of DNA methylation for a selected stretch of the DNMT3A promoter containing 17 CpGs. As shown in Fig. S3, a differential DNA methylation rate was found with methylation percentages ranging from 70% to 100% for the individual CpGs. There was a tendency toward a lower methylation rate in the CpG positions most distal (5′-upstream) to the TSS. However, no significant changes in methylation rate were observed for the 17 CpG positions when comparing DNA from the intestines of the untreated controls versus the groups exposed to 20 ppm and 200 ppm glyphosate (Fig. S3).

Aberrant DNA methylation, mediated by changes in the activity of methyltransferases. DNMT3A and DNMT3B, can affect gene expression. DNMT3A and DNMT3B are responsible for de novo methylation, which plays important roles in normal organism development and disease. Environmental factors might influence the activity of DNA methyltransferases. Tang et al. [45] studied the effects of exposure to estradiol or bisphenol A among neonatal rats. They demonstrated hypomethylation in the promoter of the nucleosome binding protein-1 (Nsbp1) but recorded no changes in DNA methylation for hippocalcin-like 1 (Hpcal1), which is a highly plastic epigenetic mark with hypermethylation that depends on both early-life exposure and events in adult life.

Omidali et al. [46] studied the promoter methylation pattern of DNMT3A and DNMT3B genes in endometrial cancer and analyzed correlations between methylation statuses with clinicopathological parameters. They found a significant difference between patients with endometrial cancer and healthy controls in terms of the presence of promoter CpG hypermethylation status in the DNMT3A gene. Furthermore, the methylation status of tissue and blood samples in the DNMT3A gene was not significant. In conclusion, the hypermethylation of the DNMT3A gene was found to be an important event in the carcinogenesis of endometrial cancer.

### 3.4 Expression analysis of IL18 mRNA

To examine whether the observed reduction in DNA methylation in the IL18 promoter (Fig. 2) had an effect on IL18 mRNA expression, we performed a quantitative RT-PCR analysis of RNA isolated from the pigs’ small intestines. Tissue samples from eight pigs from the untreated control group and eight pigs each from the groups exposed to 20 ppm and 200 ppm glyphosate were included in the expression analysis. As shown in Fig. S4, no significant changes in IL18 mRNA expression were found between the three groups. In all three experimental groups, we noticed large variation in the expression data. The small intestines were dissected according to a similar procedure to make the samples as identical as possible.

### 3.5 Expression of DNA methyltransferases

**Fig. 3:**
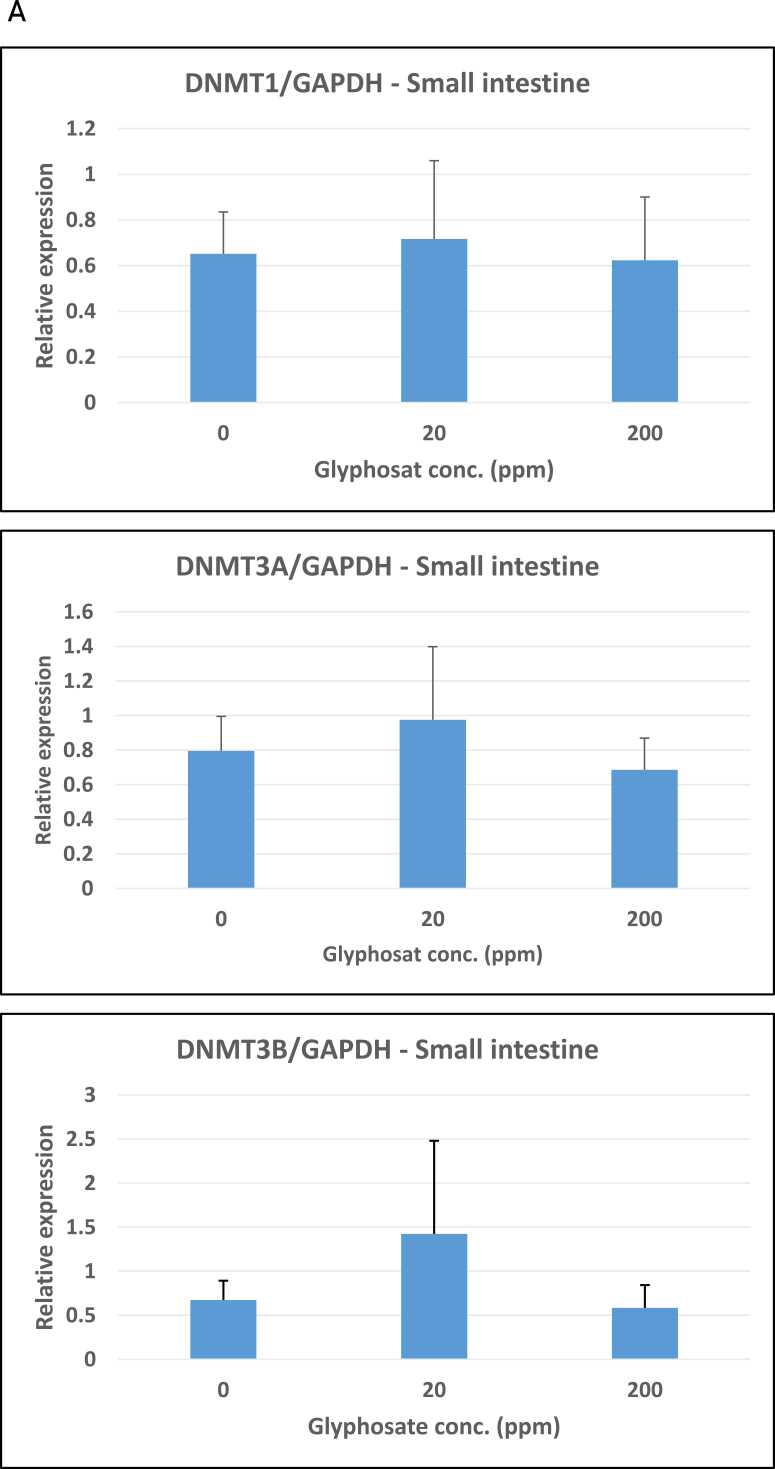

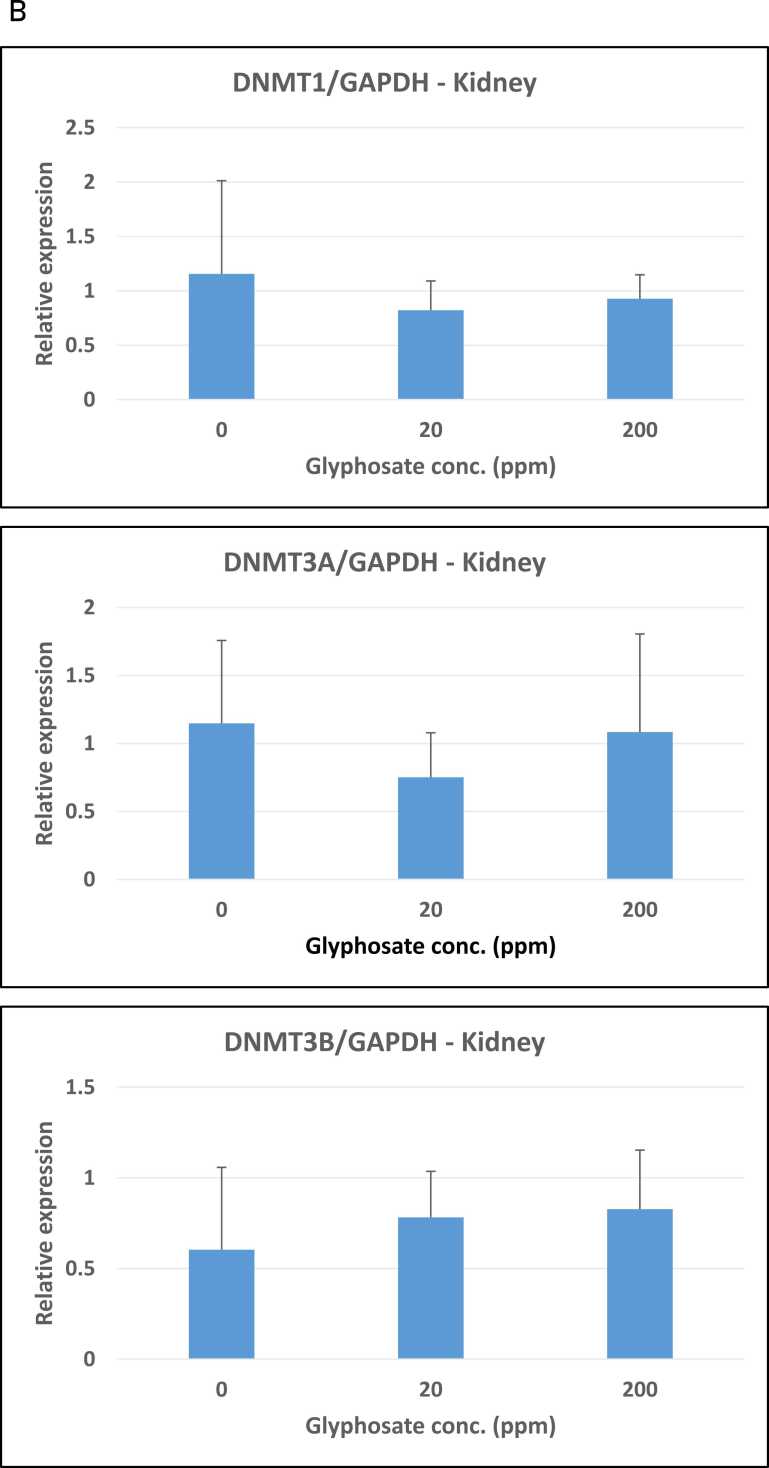

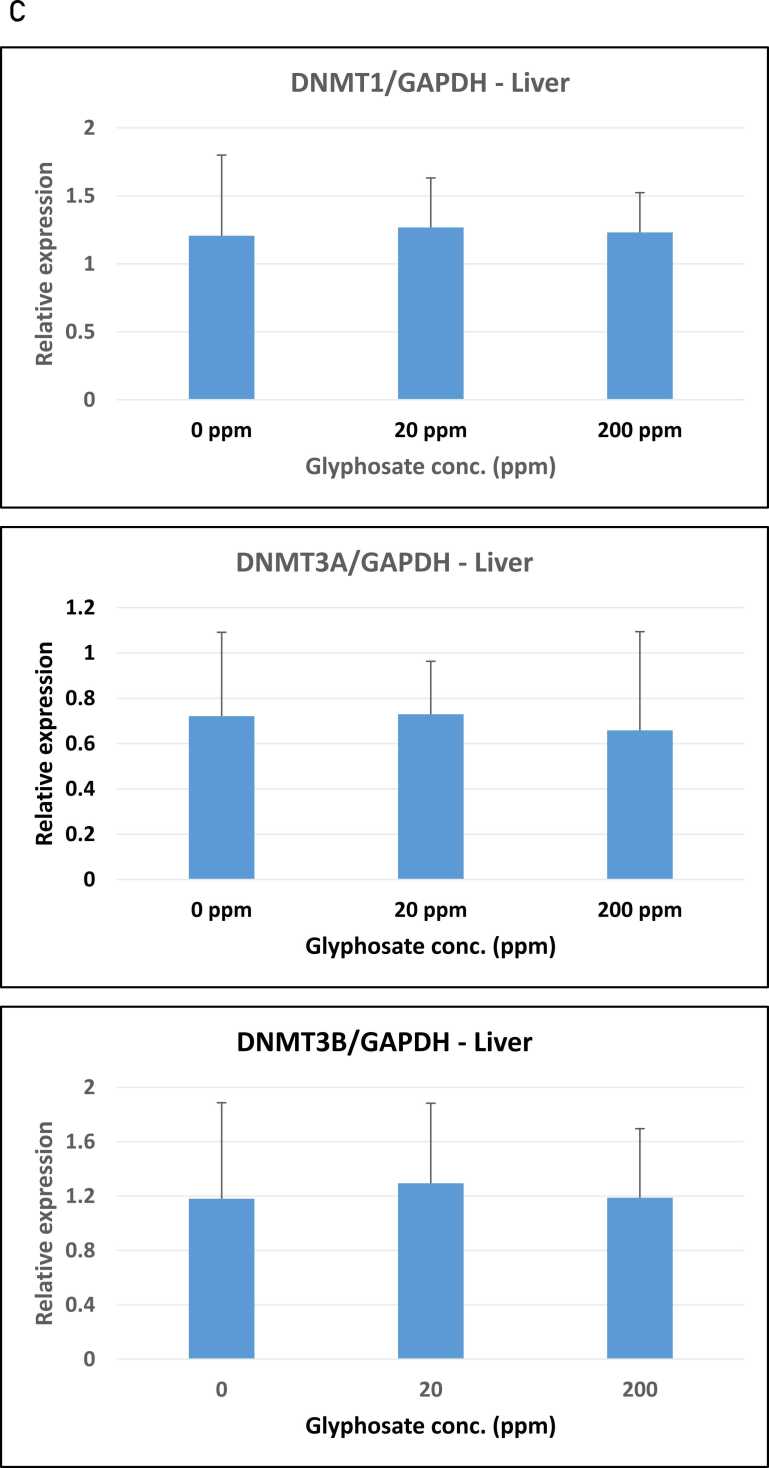
Relative expression of DNMT1, DNMT3A and DNMT3B in organs from pigs exposed to glyphosate, 0, 20, and 200 ppm. (A-C) Small intestine, (D-F) kidney, and G-I) liver. The relative expression was determined by RT-qPCR for the three experimental groups (n = 8 for each group). The analysis was performed with technical triplicates. Results are presented as bar graphs displaying the mean ± SEM. GAPDH expression was used for normalization.

The possible effect of glyphosate on the expression of the DNA methylation enzymes DNMT1, DNMT3A, and DNMT3B was examined using a quantitative RT-PCR analysis of RNA isolated from the pigs’ small intestines, kidneys, and livers. Relative expression was determined using GAPDH as the reference gene. The expression analysis demonstrated that maintenance DNA methyltransferase DNMT1 expression in the small intestine was unaffected by exposure to glyphosate (Fig. 3A-C). Similarly, the two de novo DNA methyltransferases DNMT3A and DNMT3B had almost the same mRNA expression in control pigs as compared to the pigs treated with 20 ppm and 200 ppm glyphosate (Fig. 3B and C).

Subsequent analyses showed the same expression pattern in the kidney and liver, with no significant changes in expression in untreated controls versus the groups of glyphosate-treated pigs (Fig. 3D-F and G-I).

In an earlier study, we examined the spatial expression in adult pigs and the developmental expression of DNMTs in pig embryo brains [47]. For DNMT1, DNMT3A, and DNMT3B, we observed a differential expression in the various organs and brain tissues examined.

Smith et al. [48] conducted a study with Japanese medaka (*Oryzias latipes*), where embryos (0–15 days old) were exposed to glyphosate and Roundup. A decreased expression of DNMT1 and increased expression of the methylcytosine dioxygenase genes TET1, TET2, and TET3 were observed in the developing embryos [48]. DNMT1 expression was also reduced in medaka testes in adult fish exposed to glyphosate.

Environmental factors can affect the expression of DNA methyltransferases and thereby contribute to the dysregulation of DNA methylation. This is exemplified by exposure of human keratinocytes to arsenic which induces genome-wide global DNA hypomethylation and certain specific gene promoter methylation changes. These changes can persist for many cell generations following exposure to and after withdrawal of arsenite [49]. Arsenite also induces down-regulation of DNMT3A and DNMT3B mRNA expression, while DNMT1 mRNA was unaffected. The down-regulation of DNMT3A and DNMT3B expression occurred in a dose-dependent manner and again persisted for many cell generations after the removal of the arsenite [49].

### 3.6 Expression analysis of TET3 mRNA expression

**Fig. 4:**
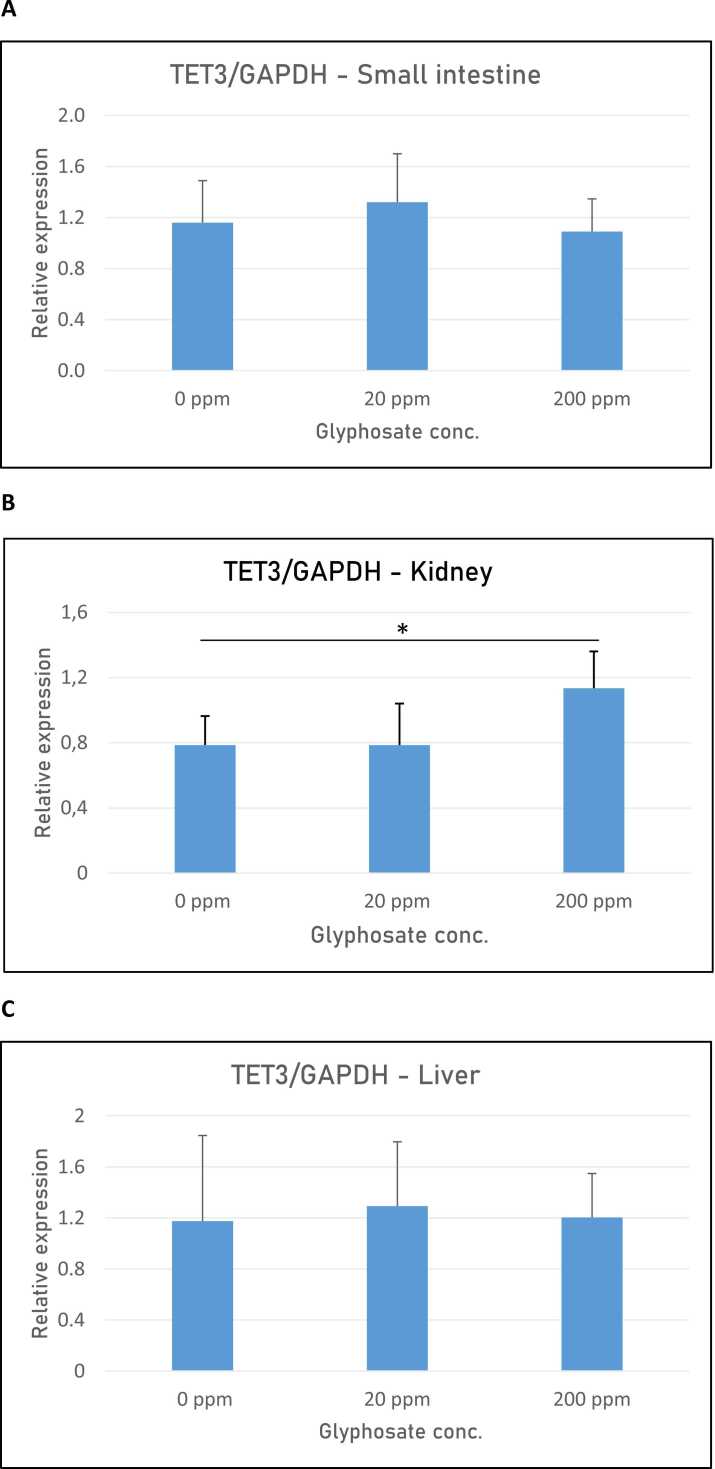
Relative expression of TET3 mRNA in organs from pigs exposed to 0, 20, and 200 ppm glyphosate. A) Small intestine, B) kidney, and C) liver. Relative expression was determined via RT-qPCR for the three experimental groups (n = 8 for each group). The analysis was performed with technical triplicates. Results are presented as bar graphs displaying the mean ± SEM. GAPDH expression was used for normalization. * P < 0.05.

In our study, we also included an expression analysis of the DNA demethylase enzyme TET3. The TET mRNA expression was determined via quantitative RT-PCR analysis of RNA isolated from the pigs’ small intestines, kidneys, and livers. No significant changes in TET mRNA expression were seen in the small intestines or liver between untreated pigs and glyphosate-exposed pigs (Fig. 4A and C). However, when examining TET3 expression in kidney tissue, we observed a significant increase (P = 0.011) in TET mRNA expression in pigs exposed to 200 ppm glyphosate compared with the untreated control group (Fig. 4B). Our results align with those of Smith et al. [48], who reported a significant increase in TET3 mRNA expression in Japanese medaka (*Oryzias latipes*) fry exposed to both glyphosate and Roundup.

Increased activity of Tet Methylcytosine Dioxygenase 3 (TET3) and a reduction in the DNA methylation of MCF10A cells exposed to glyphosate was also reported by Duforestel et al. [50]. Repeated glyphosate exposure pattern over 21 days triggered global hypomethylation and increased activity of TET3. In contrast, the increase in TET3 expression that we report in porcine kidney tissue was not accompanied by a reduction in global DNA methylation; this might be explained by the greater complexity of a whole organism compared with a cell culture and the different responses to environmental factors. TET3 is a member of Tet Methylcytosine Dioxygenase 3, also named the ten-eleven translocation, (TET) enzyme family of three dioxygenases (TET 1–3). TET enzymes convert methylcytosine into other cytosine derivatives via hydroxylation catalyzing the sequential oxidations of 5-mC to 5-hydroxymethylcytosine (5-hmC) and 5-formylcytosine.

### 3.7 CASP3 mRNA expression

Caspase 3 (CASP3) is a caspase protein cleaved into apoptotic cells that has a function in cell death and disease [51]. Hence, changes in CASP3 expression are an indicator of apoptosis.

Other research groups have measured caspase-3 mRNA expression following treatment with glyphosate [52], [53]. As such, we also included an expression analysis of the CASP3 transcript in pigs exposed to glyphosate. The mRNA expression of CASP3 was determined using a quantitative RT-PCR analysis of RNA isolated from the pig small intestines of control pigs and pigs treated with 20 ppm and 200 ppm glyphosate. No significant changes in CASP3 mRNA expression were seen in the pig intestines of untreated versus glyphosate-exposed pigs (Fig. S5). Our results are not consistent with those obtained by other groups that have studied exposure to glyphosate in both animals and cell cultures. We only examined CASP3 mRNA expression in intestines, and we therefore cannot exclude the possibility that the expression was affected by glyphosate in other tissues and organs. Ma et al. [53] reported a significant increase in caspase-9 and caspase-3 mRNA levels and activity in glyphosate-treated carp gills; this could indicate that glyphosate treatment induces mitochondria-mediated apoptosis in fish gills. A similar result of cellular apoptosis triggered by glyphosate treatment in zebrafish was obtained by Sulukan et al. [54]**.** Recently, Kwiatkowska et al. [52] reported an increase in caspase-3 activity in peripheral blood mononuclear cell PBMCs treated with 0.25 mM (~42 mg/liter) of glyphosate and the metabolites PMIDA, AMPA, hydroxymethylphosphonic acid, and bis-(phosphonomethyl)amine. Mesnage et al. [19] have performed a very thorough evaluation of the toxic and toxicogenomic effects of glyphosate of Roundup. The study was particularly focused on changes in transcriptome and epigenome profiles. Their studies included biochemical analyzes, histopathological examinations, transcriptome analyzes and DNA methylation profiling of the liver and also a selective gene expression analysis of the kidney. Mesnage et al. [19] also used mouse embryonic stem cells to study the effects of glyphosate and Roundup on DNA damage, oxidative stress, and protein folding. The histopathological and clinical biochemical analyzes documented that exposure to Roundup led to a significant increase in hepatic steatosis and necrosis. These effects were not seen upon exposure to glyphosate. The transcriptome analysis revealed that Roundup treatment altered the expression of 96 genes in the liver [19]. Among most differentially expressed genes were those related to biological functions such as TP53 activation due to DNA damage and oxidative stress and the regulation of circadian rhythms. The epigenetic (DNA methylation) profiling identified 5727 and 4496 differentially methylated CpG sites when comparing a control group of rats and groups exposed to glyphosate and Roundup. Additionally, DNA damage formation in the liver was increased with glyphosate exposure. Mechanistic studies have shown that exposure to Roundup induces oxidative stress and promotes protein folding. These effects are not seen with glyphosate. In summary, the study by Mesnage et al. shows that Roundup is more toxic than glyphosate and can have a greater effect on epigenetics and gene expression. Some of these changes may be carcinogenic.

Wozniak et al. [28] studied the effect of glyphosate on DNA methylation and gene expression of selected tumor suppressors and oncogenes in human peripheral blood mononuclear cells (PBMCs). In brief, the authors reported a significant reduction in global DNA methylation levels in PBMCs exposed to glyphosate and changed methylation levels of the P21 and TP53 suppressor gene promoters. In contrast, no changes in DNA methylation was observed in promoters for P16, BCL2, and CCND1.

Exposure of PBMCs to glyphosate also affected transcript expression of genes involved in the regulation of cell cycle and apoptosis. Glyphosate treatment decreased the expression of P16 and TP53 transcript, while an increase in the expression of BCl2, CCND1, and P21 mRNAs were seen.

## 4 Conclusion

In conclusion, the present study revealed no significant effects for exposure to dietary glyphosate concentrations (20 ppm and 200 ppm) at (e.g. soybeans) or above (e.g. wheat) EU-defined MRL for common feed crops on the global DNA methylation of weaned pigs. It is well established that the co-formulants present in glyphosate-based herbicides (GBH) are toxic in their own right (e.g. [1], [19]). Nevertheless, we did not include glyphosate from a GBH in the present study because the co-formulants vary among GBHs and are in addition usually not declared; thus results from GBH-based are not generalizable.

Similarly, most specific gene promoters did not show significant changes in DNA methylation status, and the expression of DNA methyltransferases was unaffected by glyphosate treatment. However, we found significant changes in DNA methylation in IL18 promoters, but the decrease did not affect the expression of IL18 mRNA. The other change observed in this study was the increase in TET3 mRNA expression upon treatment with glyphosate. In our study, we have focused the DNA methylation analysis on gene promoter regions. However, in future studies, it will also be relevant to investigate intragenic DNA methylation. Some intragenic DNA methylation changes have clear effects on gene expression [55], [56]. To clarify any effect of this change in DNA demethylation, we would like to extend our study to include omics analyses such as RNA-Seq and determination of the global methylome. The lack of responses of the glyphosate exposures in our measurements of DNA methylation and gene expression was surprising. We speculated, whether glyphosate contamination in the feed used in our feeding experiments have compromised our baseline measurements. We cannot rule out that a glyphosate contamination can create a saturating effect on DNA methylation and/or gene expression resulting in very small or no change in the measurement parameters. A suggested solution to this problem could be to search for animal feed, which can be proven to be completely free of glyphosate. Data from such analyses could possibly identify biomarkers and potential predictors for negative health outcomes resulting from exposure to glyphosate. The data presented by Mesnage et al. [19] demonstrates the power of high-throughput ‘omics’ methods in detecting metabolic changes and changes in epigenetics and gene expression.

## CRediT authorship contribution statement

**Martin Tang Sørensen, Ole Højberg, Knud Larsen**: Designed experiments. **Thomas Bové Christensen, Knud Larsen**: Performed the molecular experiments. **Knud Larsen**: Wrote the manuscript.

## Declaration of Competing Interest

The authors declare that they have no known competing financial interests or personal relationships that could have appeared to influence the work reported in this paper.
