# Supplementary material for: Exposure of pigs to glyphosate affects gene-specific DNA methylation and gene expression
Source: Toxicol Rep. 2022 Mar 7;9:298–310. doi: 10.1016/j.toxrep.2022.02.007 (PMC8908043; doi:10.1016/j.toxrep.2022.02.007)
Supplement: Supplementary file 1 — Supplementary material [file mmc1.docx]

A

B

Figure S1: Glyphosate-induced changes of DNA methylation status in IL18 and APEX1 promoters. A) DNA methylation was determined by bisulfite sequencing in nine CpG positions in the IL18 promoter. DNA methylation was measured in small intestine DNA from pigs exposed to 200 ppm glyphosate (orange bars) and in a control group with untreated pigs (blue bars). DNA methylation was estimated by calculating from top heights for C and T in each experimental group (n = 8 for each group). B) APEX1 - five CpG positions were analyzed for DNA methylation status. * P < 0.05.
