# Supplementary material for: Exposure of pigs to glyphosate affects gene-specific DNA methylation and gene expression
Source: Toxicol Rep. 2022 Mar 7;9:298–310. doi: 10.1016/j.toxrep.2022.02.007 (PMC8908043; doi:10.1016/j.toxrep.2022.02.007)
Supplement: Supplementary file 2 — Supplementary material [file mmc2.docx]

A

**B**

**Figure S2.** Glyphosate-induced changes of DNA methylation status in UNG and CDKN1 promoters. A) DNA methylation was determined by bisulfite sequencing in six CpG positions in the UNG promoter. DNA methylation was measured in DNA isolated from pigs exposed to 200 ppm glyphosate (orange bars) and in a control group with untreated pigs (blue bars). DNA methylation was estimated by calculating from top heights for C and T in each experimental group (n = 8 for each group). B) For the CDKN1 promoter, seven CpG positions were analyzed for DNA methylation status. Methylation rate was determined through the sequencing of individual clones (10–12) for DNA isolated from pigs exposed to 20 ppm glyphosate (orange bars), 200 ppm glyphosate (grey bars), and a control group of untreated pigs (blue bars). Three individual pigs from each experimental group were included in this study.
