# Supplementary material for: Exposure of pigs to glyphosate affects gene-specific DNA methylation and gene expression
Source: Toxicol Rep. 2022 Mar 7;9:298–310. doi: 10.1016/j.toxrep.2022.02.007 (PMC8908043; doi:10.1016/j.toxrep.2022.02.007)
Supplement: Supplementary file 3 — Supplementary material [file mmc3.docx]

**Figure S3.** Glyphosate-induced changes in DNA methylation status in the DNMT3A promoter. Seventeen CpG positions were analyzed for DNA methylation status. DNA methylation was estimated by calculating from the top heights for C and T in each experimental group (n = 8 for each group). DNA was isolated from the intestines of pigs exposed to 20 ppm glyphosate (orange bars), 200 ppm glyphosate (grey bars), and a control group of untreated pigs (blue bars). Results are presented as bar graphs displaying the mean ± SEM.
