# Supplementary material for: Exposure of pigs to glyphosate affects gene-specific DNA methylation and gene expression
Source: Toxicol Rep. 2022 Mar 7;9:298–310. doi: 10.1016/j.toxrep.2022.02.007 (PMC8908043; doi:10.1016/j.toxrep.2022.02.007)
Supplement: Supplementary file 5 — Supplementary material [file mmc5.docx]

**Figure S5.** Relative expression of caspase-3 mRNA in the small intestines of pigs exposed to 0, 20, and 200 ppm glyphosate. Relative expression was determined via RT-qPCR for the three experimental groups (n = 8 for each group). The analysis was performed with technical triplicates. Results are presented as bar graphs displaying the mean ± SEM. GAPDH expression was used for normalization.
