# Supplementary material for: Exposure of pigs to glyphosate affects gene-specific DNA methylation and gene expression
Source: Toxicol Rep. 2022 Mar 7;9:298–310. doi: 10.1016/j.toxrep.2022.02.007 (PMC8908043; doi:10.1016/j.toxrep.2022.02.007)
Supplement: Supplementary file 6 — Supplementary material [file mmc6.docx]

**Table S1**

List of oligonucleotide primers and probes used in cloning, sequencing and analysis of the porcine DNMT1, DNMT3A and DNMT3B genes.

Primer Sequence (5’ - 3’) Product size (bp) Application

APEX1-BSF2 TATTTGGAAGGAATTTTAGAAGATG 178 DNA methylation

APEX-BSR2 TAACCCACTAAACAAAAACAAAAAC DNA methylation

IL18-BSF1 TGGGAAGTTGGAGAATATTTTAGAGAA 339 DNA methylation

IL18-BSR1 ACCAAAAACCCATACTTAAAAATAATTACA DNA methylation

NEIL1-BSF1 GGTTTTTTAGTTTGGGGATTTTATT 281 DNA methylation

NEIL1-BSR1 CTACCCCCACCTATCCTAAATTAAC DNA methylation

NEIL1-BSF2 GGTTTTTGGAGAAATTAGGAATTT 181 DNA methylation

NEIL1-BSR2 AAAAAATCTACCACTTACCCCTTCT DNA methylation

NEIL1-BSF3 TTTGTTTTGGTTAAATGTTTTTTTT 213 DNA methylation

NEIL1-BSR3 AACTCCCCAAACTCCAAACTATAAT DNA methylation

NEIL2-BSF1 TGTTTTTATTAGGATAGAGTTTTTTTT 217 DNA methylation

NEIL2-BSR1 TAAACCCTTAAACCCCAACAAC DNA methylation

NEIL2-BSF2 AAAAATTTATTTTTGAAAAATTTTA 196 DNA methylation

NEIL2-BSR2 CCCTTAAAAAAATCATTATCTTATAC DNA methylation

OGG1-BSF1 GGATAGTGAGGATTAGGGTTTGAG 235 DNA methylation

OGG1-BSR1 TAATTAAATCCAAAAATAACCAATC DNA methylation

UNG-BSF2 TTTTTGTTTTTTGTTTTTTTAGGGT 194 DNA methylation

UNG-BSR2 TACAAATTCAATCCCTAATCTCCAT DNA methylation

POLB-BSF1 TATTGGGATGTAATTTTGGGGTAT 242 DNA methylation

POLB-BSR1 AAACAATAACTACCTCTAACAAAAAAC DNA methylation

POLB-BSF2 TATTGGGATGTAATTTTGGGGTAT 240 DNA methylation

POLB-BSR2 ACAATAACTACCTCTAACAAAAAAC DNA methylation

DNMT3A-BSF TGATTGTATTAGAAAATAATGGAAGGA DNA methylation

DNMT3A-BSR CATATAAAAATTCCCAAACTAAAAATTAAAT DNA methylation

CDKN1A-BS2F TGGTTTAGTGGATAGTGAGTAGTTG 165 DNA methylation

CDKN1A-BS2R CCAAAAAAATCTCAATAATAAAATC DNA methylation

**P14ARF-BS3F** GTTTGTGGGGGCGGGGATGGGTA 242 DNA methylation

P14ARF-BS3R GCTAACTCCTCACTAACAACAACACG DNA methylation

DNMT1-RTF GACTCAGAAGTCAAACCAAAGAACTAACACC 171 Expression

DNMT1-RTR TTTCTTCAGGTCTCCGTTTGCCAGC Expression

DNMT1-probe TGGAGGG (#25*)

DNMT3A-RTF CTGAGAAGCCCAAGGTCAAGGA 167 Expression

DNMT3A-RTR GCAGTTTTGGCACATTCCTCCGAT Expression

DNMT3A-probe GGCAGAAG (#29*)

DNMT3B-QF CAGACAACTCAACGGAGAGGA 80 Expression

DNMT3B-QR CCCATTGGTGATGATGGAGT Expression

DNMT3B-probe CAGCAGGA (#18*)

TET3-RTF CAGAGAGCCTGGCAAGACA 60 Expression

TET3-RTR GGTCCCCCGTTCTGAGAT Expression

TET3-probe CTCTGCCT (#13*)

IL18-QF ACTTTACTTTGTAGCTGAAAACGATG 67 Expression

IL18-QR TTTAGGTTCAAGCTTGCCAAA Expression

IL18-probe GACCTGGA (#85*)

CASP3-QF TGCATATTC TACAGCACCTGGTT 60 Expression

CASP3-QR AACCAGGATCCGTCCTTTG Expression

CASP3-probe TTCCTGGC (#72*)

GAPDH-F TGGTGAAGGTCGGAGTGA Expression

GAPDH-R GGCCATCCACAGTCTTCTGAG 564 Expression

GAPDH-probe CATCACCA (#9*)

*Probes are from the Roche Human Probe library
